# Risk factors associated with an increase in the size of ground‐glass lung nodules on chest computed tomography

**DOI:** 10.1111/1759-7714.13098

**Published:** 2019-06-02

**Authors:** Hee‐Young Yoon, Ji‐Yun Bae, Yookyung Kim, Sung Shin Shim, Sojung Park, So‐Young Park, Soo Jung Kim, Yon Ju Ryu, Jung Hyun Chang, Jin Hwa Lee

**Affiliations:** ^1^ Department of Internal Medicine, College of Medicine Ewha Womans University Seoul Korea; ^2^ Department of Radiology, College of Medicine Ewha Womans University Seoul Korea

**Keywords:** Ground glass lung nodules, low‐dose computed tomography, lung cancer, prognosis, screening

## Abstract

**Background:**

The detection rate of ground‐glass nodules (GGNs) in the lung has increased with the increased use of low‐dose computed tomography (CT) of the chest for cancer screening; however, limited data is available on the natural history, follow‐up, and treatment of GGNs. The aim of this study was to identify factors associated with an increase in the size of GGNs.

**Methods:**

A total of 338 patients (mean ages, 59.8 years; males, 35.5%) with 689 nodules who underwent chest CT at our institute between June 2004 and February 2014 were included in this study. The cut‐off date of follow‐up was August 2018. We analyzed the size, solidity, number, and margins of the nodules compared with their appearance on previous chest CT images. The Cox proportional hazard model was used to identify risk factors associated with nodule growth.

**Results:**

The median follow‐up period was 21.8 months. Of the 338 patients, 38.5% had a history of malignancy, including lung cancer (8.9%). Among the 689 nodules, the median size of the lesions was 6.0 mm (IQR, 5–8 mm), and the proportion of nodules with size ≥10 mm and multiplicity was 17.1% and 66.3%, respectively. Compared to the nodules without an increase in size, the 79 nodules with an increase in size during the follow‐up period were initially larger (growth group, 7.0 mm vs. non‐growth group, 6.0 mm; *P* = 0.027), more likely to have a size ≥10 mm (26.6% vs. 15.9%; *P* = 0.018), and had less frequent multiplicity (54.4% vs. 67.9%, *P* = 0.028). In the multivariate analysis, nodule size ≥10 mm (hazard ratio [HR], 2.044; *P* = 0.005), a patient history of lung cancer (HR: 2.190, *P* = 0.006), and solitary nodule (HR: 2.499, *P* < 0.001) were independent risk factors for nodule growth.

**Conclusion:**

Careful follow‐up of GGNs is warranted in patients with a history of malignancy, a large , or a solitary nodule.

## Introduction

Lung cancer shows the highest incidence and mortality of all cancers worldwide, which is attributed to the relatively high proportion of patients with advanced stage disease at the time of diagnosis.^1^ The development and study of screening tests for early detection of lung cancer has steadily advanced, and low‐dose chest computed tomography (CT) has proved efficacious for reducing lung‐cancer related mortality in selective populations.^2^, ^3^, ^4^ Accordingly, several guidelines including those of the National Comprehensive Cancer Network, American College of Chest Physicians, American Thoracic Society, American Association for Thoracic Surgery, and American Cancer Society recommend annual low‐dose chest CT for lung cancer screening in high‐risk individuals.^5^, ^6^, ^7^, ^8^, ^9^ The incidence of pulmonary nodules is rising with the increasing development of low‐dose CT technology and frequency of its use for cancer screening.

A ground‐glass nodule (GGN) in the lung is defined as a hazy area of increased pulmonary attenuation with preservation of the bronchial and vascular margins. Newly‐detected GGNs reportedly occur in 0.7% (485/64 677) of the screened population per year, resulting in the clinical diagnosis of various benign and malignant conditions.^10^ Although GGNs may disappear or decrease in size by 50% within threemonths, those that persist longer than threemonths are more likely to be malignant or premalignant lesions, such as atypical adenomatous hyperplasia (AAH) and adenocarcinoma in situ (AIS).^11^ In particular, mixed GGNs (those containing a solid portion) have a high likelihood of malignancy.^11^, ^12^, ^13^ However, those GGNs that remain unchanged in size over time are much less likely to be malignant.^14^, ^15^ While observation may be an appropriate approach for some GGNs, it is important to identify rapidly progressing GGNs for biopsy. In addition, lung cancer derived from slow growing GGNs does not significantly affect an individual's death,^13^, ^16^ while aggressive treatment such as surgical resection should be considered for rapidly growing GGNs. Although several studies have previously reported the risk factors associated with an increase in GGN size,^12^, ^17^, ^18^, ^19^, ^20^, ^21^ the sample sizes were small and the follow‐up periods relatively short. The aim of this study was to identify factors associated with an increase in the size of newly‐detected GGNs in a large study population with a long follow‐up period.

## Methods

### Study population

**Figure 1:**
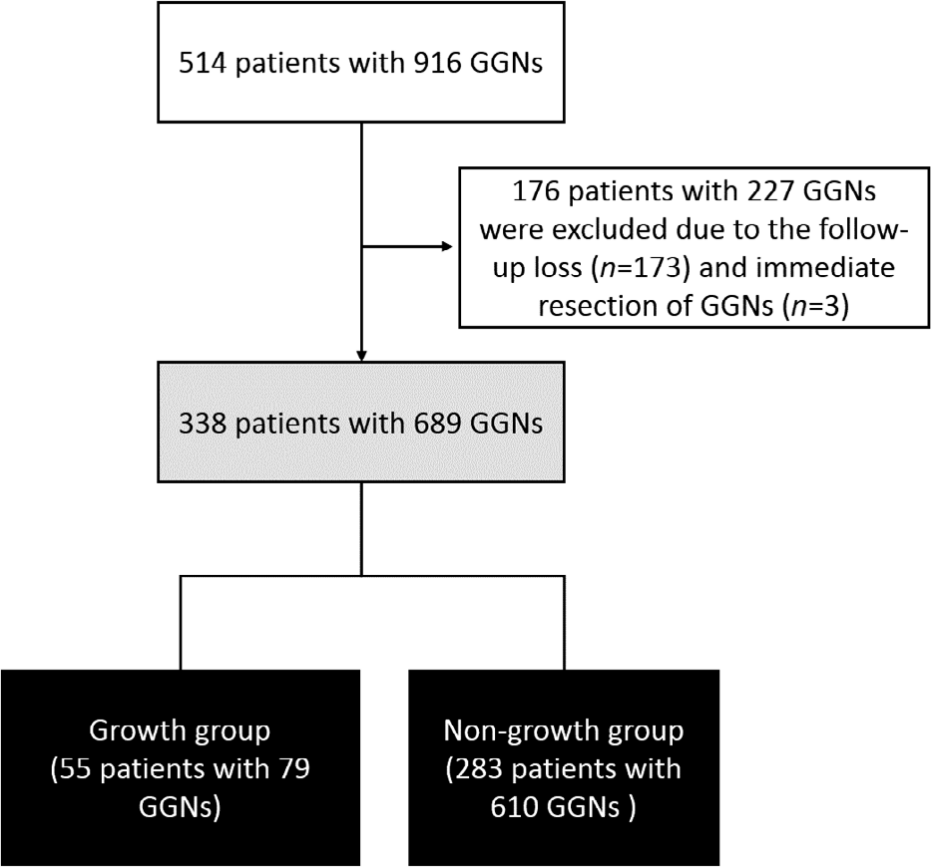
Enrollment of patients. GGNs, ground‐glass nodules.

Five‐hundred and fourteen patients with 916 GGNs detected on chest CT were initially screened at Ewha Womans University Mokdong Hospital (Fig 1) between June 2004 and February 2014. A total of 338 patients with 689 GGNs who were available for follow‐up chest CT and did not undergo lung resection of the GGNs were included in our study. Patients without follow‐up data for GGNs were excluded due to: (i) resected GGNs (*n* = 3) immediately after the GGN detection, and (ii) follow‐up loss (*n* = 173). A GGN was defined as a circumscribed area of ground‐glass opacity with preservation of the bronchial and vascular margins. All subjects were divided into growth and non‐growth groups according to whether the size of GGN increased. Informed consent was waived due to the retrospective study design, and this study was approved by the institutional review board of Ewha Womans University Medical Center (EUMC‐2019‐02‐001).

### Study design and radiological finding evaluation

The data on demographics, medical history, and radiological findings at the time of first GGN detection were retrospectively obtained through medical charts. Overall cancer history, which was defined as a diagnosis of cancer before our study enrollment and lung cancer history, were analyzed separately from overall cancer history. Underlying lung disease was a radiologic abnormal finding at time of initial chest CT. Chest CT scans included images of routine enhanced CT, high‐resolution CT, and low‐dose CT with slice thickness of 3 mm (from 2004 to 2012) or 2 mm (from 2013 to 2018). The characteristics of the GGNs (size, number, margin type, and solidity) were collected through review of the CT scan from the time of first GGN detection. The nodule size (in mm) was defined as the longest diameter, measured with electronic calipers. The GGNs were compared by dividing them into two groups based on their sizes (equal to, or larger than, 10 mm, or smaller than 10 mm), number (single or multiple), margin (irregular or regular, ill‐defined or well‐defined), and solidity (presence or absence). A GGN with an irregular margin was defined as a GGN without a smooth border. A GGN with an ill‐defined margin was defined as a GGN where it is difficult to determine the boundary line. The characteristics of the GGNs were interpreted by two experienced thoracic radiologists by consensus in cases of disagreement.

The GGN was followed‐up at intervals of 3–12 months under the Fleischner Society recommendation, and modified according to an individual's past history.^22^ We reviewed the follow‐up chest CT images obtained after the first image showing GGN detection to investigate the time of the changes in radiological features (namely, increase in size, number, solidity, and margin type) from enrollment to August 2018. The follow‐up time for a nodule with increasing size was defined as the period between the date of the first appearance of the nodule on chest CT and the date of the CT showing a size increase, while that for a nodule with no change in size was defined as the period between the initial detection of the GGN to the last follow‐up chest CT.

### Statistical analysis

Continuous variables were expressed as mean ± standard deviation (SD) or median (interquartile range, IQR). Categorical variables were presented as number (%). For comparison between two groups, Student's *t*‐test or the Mann Whitney U test was used for continuous variables and the Chi‐square test or Fisher exact test was used for categorical variables. Survival rates with 95% confidence intervals (CI) were analyzed using the Kaplan‐Meier method, and the differences between groups were compared with the use of the log‐rank test. The risk factors for increasing size of GGNs were identified using Cox proportional regression analysis and expressed as a hazard ratio (HR) with 95% CI. Covariates with *P* < 0.1 on univariate analysis were included in the multivariate analysis. A *P*‐value <0.05 was considered significant (two‐tailed). All statistical analysis was performed using SPSS version 23.0 (SPSS Inc., Chicago, IL, USA).

## Results

### Baseline demographics

The mean age of the 338 patients with GGNs (Fig 1) was 59.8 years, 35.5% were male, and 10.8% were ever‐smokers (Table 1). One‐hundred‐thirty patients (38.5%) had a history of cancer, including lung cancer (8.9%) and breast cancer (7.7%). The most common underlying lung disease was lung cancer (12.4%), followed by bronchiectasis (11.2%) and pneumonia (7.5%).

**Table 1:** Baseline characteristics of patients with ground‐glass nodules

Characteristics	
Number	338
Age, years	59.8 ± 11.6
Male sex	120(35.5)
Ever smoker	26/240(10.8%)
Smoking amount, pack–years	22.3 ± 16.3
History of malignancy	130(38.5)
Lung cancer	30(8.9)
Breast cancer	26(7.7)
Thyroid cancer	18(5.3)
Lymphoma	12(3.6)
Underlying lung diseases	
Lung cancer	42(12.4)
Bronchiectasis	38(11.2)
Pneumonia	22(6.5)
Tuberculosis sequelae	21(6.2)
Emphysema	14(4.1)

Data are presented as mean ± SD or number (%), unless otherwise indicated.

### Radiological features of the GGNs

The median size of the 689 GGNs at initial CT scans was 6.0 mm (IQR, 5.0–8.0 mm) (Table 2); 17.1% were larger than 10 mm, 66.3% were multiple GGNs, 3.6% contained the solid portion, and 9.3%, and 0.8% had ill‐defined and irregular margins, respectively. During the follow‐up period (median, 21.8 months; IQR, 6.1–54.9 months), 79 GGNs (11.5%) increased in size, 61 (8.9%) revealed development of multiplicity, and 26 (3.8%) acquired new solid portions.

**Table 2:** Radiologic characteristics of ground glass nodules at initial chest CT scans

Characteristics	
Number	689
Lesion size, mm	6.0(5.0–8.0)
Longest diameter ≥ 10 mm	118(17.1)
Multiple GGN	457(66.3)
Ill‐defined margin	64(9.3)
Irregular margin	7(0.8)
Presence of internal solid portion	25(3.6)
Changes during follow‐up	
Increase in size	79(11.5)
Multiplicity	61(8.9)
Solid portion	26(3.8)
Margin	1(0.1)
Median follow‐up duration, months	21.8(6.1–54.9)

Data are presented as median (IQR) or number (%), unless otherwise indicated.

GGN, ground glass nodule; IQR, interquartile range.

### Comparison of baseline patient characteristics and radiological features between GGNs with and without size increase

Seventy‐nine GGNs (11.5%) in 55 patients (16.3%) showed an increase in size during the follow‐up period (Fig 1). Compared to the patients whose GGNs did not increase in size, the 55 patients with growing GGNs more often had a history of cancer (growth group, 58.2% vs. non‐growth group, 34.6%, *P* = 0.001), including lung (20.0% vs. 6.7%, *P* = 0.004) and thyroid cancer (12.7% vs. 3.9%, *P* = 0.015). In addition, patients with GGN growth were more likely to have lung cancer (21.8% vs. 10.6%, *P* = 0.021) (Table 3). Compared to the non‐growing GGNs, the growing GGNs exhibited large initial nodule size (growth group, 7.0 mm vs. non‐growth group, 6.0 mm; *P* = 0.027), less multiplicity (54.4% vs. 67.9%, *P* = 0.024), and a lower frequency of ill‐defined margins (2.5% vs. 10.2%, *P* = 0.028; Table 4). The proportion of GGNs showing solidity and irregular margins were not different between the growth and non‐growth groups.

**Table 3:** Comparison of baseline characteristics between patients with increased ground‐glass nodules and those without

Characteristics	Patients with growth	Patients without growth	*P*‐value
Number	55	283	
Age, years	60.2 ± 10.7	59.7 ± 11.8	0.494
Male sex	24(43.6)	96(33.9)	0.168
Ever smoker	4/43(9.4)	22/197(11.3)	1.000
Smoking, pack–years	19.4 ± 14.8	22.8 ± 16.9	0.705
History of cancer	32(58.2)	98(34.6)	0.001
Lung cancer	11(20.0)	19(6.7)	0.004
Breast cancer	3(5.5)	23(8.1)	0.781
Thyroid cancer	7(12.7)	11(3.9)	0.015
Lymphoma	2(3.6)	10(3.5)	1.000
Underlying lung disease			
Lung cancer	12(21.8%)	30(10.6%)	0.021
Bronchiectasis	2(3.6)	36(12.7)	0.051
Pneumonia	1(1.8)	21(7.4)	0.226
Tuberculosis sequelae	3(5.5)	18(6.4)	1.000
Emphysema	1(1.8)	13(4.6)	0.345

Data are presented as mean ± SD or number (%), unless otherwise indicated.

**Table 4:** Comparison of radiologic characteristics between increased and non‐increased ground‐glass nodules

Characteristics	Growth	Non‐growth	*P*‐value
Number	79	610	
Lesion size, mm	7.0(5.0–10.0)	6.0(5.0–8.0)	0.027
Longest diameter ≥ 10 mm	21(26.6)	97(15.9)	0.018
Multiple GGN	43(54.4)	414(67.9)	0.024
Ill‐defined margin	2(2.5)	62(10.2)	0.028
Irregular margin	1(1.3)	6(1.0)	0.575
Presence of solid portion	4(5.1)	21(3.4)	0.516

Data are presented as median (IQR) or number (%), unless otherwise indicates.

GGN, ground‐glass nodule; IQR, interquartile range.

### Risk factors for increases in GGN size

**Figure 2:**
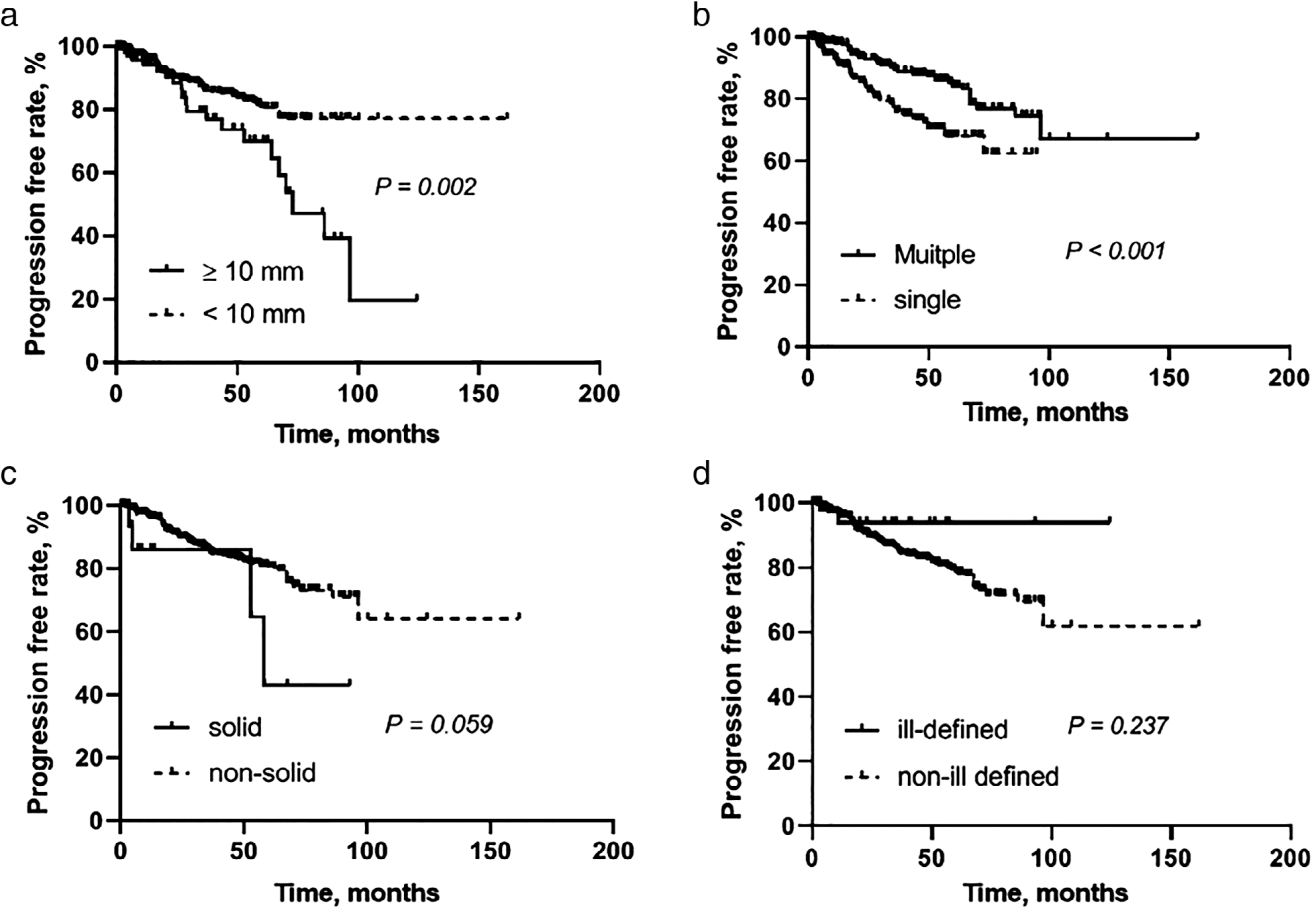
Kaplan‐Meier curves of increases in ground‐glass nodules size according to nodule size (**a**), multiplicity (**b**), the presence of a solid portion (**c**) and ill‐defined margin (**d**)

The median time from diagnosis to size increase was 19.7 months (IQR, 5.9–50.1 months). The three‐ and five‐year progression rates were 14.2%, and 18.9%, respectively. There was significantly more progression in GGNs larger than 10 mm than in those smaller than 10 mm (*P* = 0.002, Fig 2a). Also, size increase occurred less often in multiple GGNs than in solitary GGNs (*P* < 0.001, Fig 2b). A non‐significant trend toward size increase was observed in GGNs with a solid portion compared to those without a solid portion (*P* = 0.059, Fig 2c). In contrast, the GGNs with ill‐defined margins did not significantly differ in progression rate compared with GGNs without ill‐defined margins (*P* = 0.237, Fig 2d).

Univariate Cox analysis demonstrated that history of malignancy, history of lung cancer, larger initial GGN size, size larger than 10 mm, and solitary GGNs were associated with an increase in size (Table 5). On multivariate analysis, which included variables with *P* < 0.1, history of lung cancer (HR, 2.190; 95% CI, 1.253–3.826; *P* = 0.006), large size (size ≥10 mm; HR, 2.044; 95% CI, 1.235–3.382; *P* = 0.005), and a single nodule (HR, 2.499; 95% CI, 1.540–4.057; *P* < 0.001) were independent predictors for size increasing, while a history of thyroid cancer showed an association trend with size increase (HR, 1.839; 95% CI, 0.972–3.479; *P* = 0.061) (Table 5).

**Table 5:** Univariate and multivariate analyses for factors associated with increase in sizes of ground‐glass nodules

	Univariate			Multivariate		
Variable	Hazard ratio	95% confidence interval	*P*‐value	Hazard ratio	95% confidence interval	*P*‐value
Age, years	1.012	0.993–1.031	0.209			
Female	0.714	0.457–1.118	0.141			
Ever smoker	1.354	0.618–2.967	0.449			
History of malignancy	1.923	1.218–3.035	0.005			
Lung cancer	2.782	1.652–4.684	<0.001	2.190	1.253–3.826	0.006
Thyroid cancer	1.647	0.945–2.870	0.078	1.839	0.972–3.479	0.061
Pneumonia	0.602	0.148–2.456	0.480			
Bronchiectasis	0.417	0.131–1.321	0.137			
Tuberculosis	1.765	0.709–4.394	0.222			
Emphysema	0.272	0.038–1.954	0.195			
Lung cancer	1.324	0.785–2.232	0.292			
Lesion size, mm	1.086	1.037–1.137	<0.001			
Longest diameter ≥10 mm	2.153	1.306–3.548	0.003	2.044	1.235–3.382	0.005
Solitary nodule	2.232	1.426–3.494	<0.001	2.499	1.540–4.057	<0.001
Solidity	2.545	0.930–6.966	0.069	2.383	0.848–6.702	0.100
Irregular margin	0.801	0.111–5.774	0.826			
Ill‐defined margin	0.439	0.108–1.790	0.251			

## Discussion

Our study which involved 338 patients with 689 GGNs demonstrated an increase in size in 11.5% of the GGNs, with a median time to size increase of 19.7 months. Patient history of cancer (including lung and thyroid cancer), large nodule size, and solitary GGN were independent risk factors for GGN growth.

In our study, large size at the time of detection was significantly associated with an increase in GGN size; this is in accordance with previous research showing that nodule size is an important prognostic factor. The guidelines from the Fleischner Society 2017 and the British Thoracic Society base the recommended follow‐up CT interval and tissue sampling according to the size of nodules and the risk of lung cancer.^23^, ^24^ In several lung cancer risk prediction models, the lung nodule size is an essential component.^25^, ^26^, ^27^ Specifically, several studies have reported that GGNs larger than 10 mm are significantly associated with outcomes.^17^, ^18^, ^28^, ^29^, ^30^ A study reported by Lee *et al*. revealed that in pure GGNs, large size (≥ 10 mm) was a significant risk factor for size increase (odds ratio [OR], 6.46; 95% CI, 2.7–15.6) in 114 patients with 175 GGNs.^18^ In addition, Matsuguma *et al*. in their study involving 98 pure GGNs in patients with a history of lung cancer, observed that nodule size greater than 10 mm was an independent risk factor for nodule growth (HR, 13.7; *P* = 0.001).^28^ In a patient cohort in Japan, similar findings were reported by Sato *et al*. showing that large size (≥ 10 mm) at initial diagnosis (OR, 43.6; 95% CI, 6.01–998) was associated with nodule growth at 36 months in 78 patients with multiple GGNs (*n* = 299).^17^ These results are in accordance with our findings. Therefore, we conclude that GGNs larger than 10 mm at the time of detection may require more aggressive management and short‐term follow‐up rather than observation. In addition, a history of lung cancer, as shown in our study and others, is a risk factor for GGN growth.^12^, ^17^, ^29^ Therefore in patients with large GGNs and a history of lung cancer, treatment should also be managed aggressively.

According to our study, the presence of a solid portion was not associated with GGN growth, which differs from the results of previous studies.^18^, ^20^, ^21^, ^28^, ^29^ Kim *et al*. showed that in 92 patients who underwent follow‐up chest CT after resection, having a solid portion (OR, 41.8; 95% CI, 7.710–379.6) was associated with increasing GGN size on multivariate analysis^21^; similar findings were reported by Lee *et al*. (OR for GGN growth, 2.69; 95% CI, 1.11–6.95).^18^ Chang *et al*. also demonstrated that benign GGNs less frequently had a solid component (benign [*n* = 20], 3.7% vs. malignant [*n* = 39], 26.0%, *P* < 0.001) than malignant GGNs in patients with extrapulmonary cancers.^33^ The lack of significance of the presence of a solid portion in our study might be attributed to the diagnostic resection without observation of such nodules due to their high risk of malignancy, precluding confirmation of nodule size increase.^13^, ^31^ Also, the proportion of patients undergoing lung resection after the initial detection of GGNs with a solid portion might be relatively high in our study compared to previous studies, as more knowledge of the association between the presence of a solid portion and malignant potential has accumulated. However, the recent study by Sato *et al*. showed that a part‐solid GGN was only a significant factor for GGN growth in multiple GGNs in the univariate analysis and not in the multivariate analysis,^17^ similar to the association observed in our study.

In our study, single nodules had a higher risk of growth than multiple nodules. It remains controversial whether single or multiple nodules have worse outcomes. The number of lung nodules was negatively associated with the diagnosis of lung cancer (OR, 0.92; 95% CI, 0.82–1.00; P = 0.048) in 1090 persons (5021 nodules) who underwent screening low‐dose chest CT.^25^ In contrast, Kim *et al*. revealed that multiple pure GGNs (median size, 5 mm) did not show an increase in size during the observation period in 73 patients who underwent lung resection for bronchioalveolar carcinoma.^32^ However, in other reported studies, the number of nodules was not related to an increase in GGN size.^20^, ^21^, ^33^ In our study, it is possible that the large size of the solitary nodules (median, 7 mm [solitary] vs. 6 mm [multiple], *P* < 0.001) might have contributed to their elevated risk for growth. The observation by Kim *et al*. that multiple GGNs were smaller in average size than single GGNs (12 ± 7.9 mm [multiple] vs. 17 ± 8.1 mm [solitary], *P* < 0.001)^34^ also supports our findings.

We demonstrated that the GGNs progression course was indolent with a median time from GGNs detection to growth of 19.7 months. Most type of GGNs identified as adenocarcinoma have lepidic predominant patterns with good prognosis and slow progression.^35^ Takahashi *et al*. revealed that lepidic growth predominant adenocarcinoma sized 3–5 cm showed excellent prognosis compared with non‐lepidic predominant patterns adenocarcinoma.^36^ Araki *et al*. also reported that lepidic growth adenocarcinoma was less invasivene than non‐lepidic growth adenocarcinoma on histological examination.^37^ Our study did not perform histological evaluation of increased GGN, but an indolent course of GGNs supported the fact that relatively good prognostic lung cancer such as lepidic growth pattern might be associated with GGNs.

Our study had some limitations. First, because this was a retrospective study, there may have been some errors in comparing the radiological features due to non‐uniform CT imaging protocols. In particular, the size of nodule in thicker CT sections may have been underestimated compared to that in thin CT sections. However, a 1 mm difference between the thick and thin sections may not have significantly influenced our result. Second, the volume of the actual three‐dimensional nodule may not be reflected because we analyzed the longest diameter of the nodule. Third, the CT scan interval was not constant, so it was limited to accurately detecting when the GGN size increase occurred. Lastly, the follow‐up period may have been insufficient to detect the progression of slow‐growing cancer, particularly for lepidic adenocarcinoma. Despite these limitations, an advantage of this study was the inclusion of a large number of GGNs. Also, because we targeted GGNs detected in all CTs performed within a specific period, the underlying diseases in the study subjects varied. Thus, we could identify links between clinical characteristics, such as cancer history, and the increases in GGN size.

In conclusion, solitary GGNs which are large in size at initial diagnosis and GGNs in patients with a history of lung cancer were significantly associated with an increase in nodule size. Therefore, GGNs with these characteristics should be examined histologically at the time of detection rather than being observed over a period of time. In addition, considering the median progression time of 19.7 months in our study, at least two years of intensive follow‐up may be required for GGNs with a high risk of growth.

## Author contributions

Jung Hyun Chang was responsible for the conception and design of the study. Jung Hyun Chang provided study materials, patients and administrative support. Hee‐Young Yoon, Ji‐Yun Bae, and Jung Hyun Chang collected and assembled the data. Hee‐Young Yoon, Ji‐Yun Bae, Yookyung Kim, and Jung Hyun Chang analysed and interpreted the data. All authors were responsible for writing the manuscript and gave their final approval for its publication.

## Funding

This study is supported by the National Research Foundation of Korea Grant funded by the Korean Government (2010‐0027945) and Research foundation of EWHA Medical Alumni Association from EWHA Medical Alumni Association.

## Disclosure

Not declared

Financial Disclose: Not declared.
